# Grand challenges in cell death and survival: apoptosis vs. necroptosis

**DOI:** 10.3389/fcell.2014.00003

**Published:** 2014-02-20

**Authors:** Craig M. Walsh

**Affiliations:** Department of Molecular Biology and Biochemistry, Multiple Sclerosis Research Center, Institute for Immunology, University of CaliforniaIrvine, Irvine CA, USA

**Keywords:** cell death, apoptosis, necrosis, necroptosis, Grand Challenges, caspases

Cell death is, perhaps paradoxically, essential for life. This is particularly so for multicellular organisms, where cell death plays crucial roles in regulating embryonic development, tissue homeostasis, immune function, tumor suppression, and infection resistance. Much of what is known about cell death has been developed through studies in the last two decades, an era that has witnessed an explosion of publication in the area of programmed cell death (PCD). In this Grand Challenges monograph, I provide a short background on what we've learned thus far. I will then highlight areas that are likely to be of strong focus in the future, focusing primarily on distinct forms of PCD including caspase-dependent apoptosis, programmed necrosis/necroptosis, and autophagic cell death. The relevance of these cell death pathways to disease is described, and the potential that manipulation of these pathways may be the key to treating such diseases is considered. My intent is not to provide an encyclopedic review of the field, a task that would likely fill many volumes. Instead, I wish to highlight areas that are likely to be relevant to our mission as we christen a new Frontiers Specialty area, Frontiers in Cell Death and Survival.

## Caspase-dependent apoptosis

Perhaps the best-studied form of cell death is apoptosis, a genetically programmed mechanism for promoting the orderly demise of cells upon receipt of a death stimulus, or via failure to receive survival signals. First described morphologically in the context of dying thymocytes (Kerr et al., 1972), apoptosis has been extensively characterized over the last two decades. These studies have revealed complex apoptotic mechanisms that are interwoven with cell cycle, cellular metabolic, and receptor signal transduction pathways. Apoptotic cell death is primarily engaged in eliminating damaged or stressed cells in a manner that is likely to induce the least tissue damage and local inflammation (Garg et al., 2010). Central to the molecular events that unfold during apoptosis, caspases are a family of cysteine proteases with specificity for aspartate residues present on protein substrates (Thornberry and Lazebnik, 1998). These caspases specifically cleave a variety of proteins involved in cellular physiology, including those that modulate DNA fragmentation, proteins that regulate the externalization of the phospholipid phosphatidyl-serine on the plasma membrane (thus serving as an engulfment signal), DNA repair proteins such as poly-(ADP-ribose) polymerase (PARP) and signaling proteins required for cell survival and energy metabolism (Elkon, 1999).

Caspases were originally discovered by their involvement with cytokine processing; indeed, caspase-1 was originally termed interleukin-1 beta converting enzyme (ICE) due to its ability to cleave and process cytosolic pro-interleukin-1 beta (Cerretti et al., 1992; Thornberry et al., 1992). Caspases were subsequently found to contribute to apoptotic cell death by Horvitz, Yuan and colleagues (Miura et al., 1993; Yuan et al., 1993), and there are two major families of these proteases involved in apoptotic signaling, apical, and executioner caspases (Thornberry and Lazebnik, 1998). Apical caspases are typically activated by dimerization with adaptor proteins that promote their activation by induced proximity (Salvesen and Dixit, 1999). Apical caspases include caspases-8 and -10 that are activated by binding of tumor necrosis factor (TNF) family ligands such as CD95/Fas ligand, TNF alpha and TRAIL (Ashkenazi and Dixit, 1998), and caspase-9 which is activated by the release of cytochrome C from mitochondria (Green and Reed, 1998). Executioner caspases include caspases-3, -6, and -7, and these proteases attain activity primarily through cleavage by apical caspases (Pop and Salvesen, 2009). Since the process involves a cascade of cleavage events, apoptotic signals are thought to be amplified beyond a “point of no return,” thus leading to definitive cell death events (Kroemer et al., 1995).

Two major forms of apoptosis have emerged: (1 intrinsic, which involves the release of cytochrome C from mitochondria to the cytoplasm with the activation of apical caspase-9, and b) extrinsic, which involves the binding of extracellular TNF family ligands to death receptors, with the subsequent activation of apical caspases-8 and -10 (Yuan, 1997). Intrinsic apoptosis is regulated by proteins of the Bcl-2 family, which itself includes both pro- and anti-apoptotic members. These Bcl-2 family proteins modulate the release of cytochrome C under a variety of cellular conditions, and are subject both transcriptional and post-translational regulation (Chipuk et al., 2010). Extrinsic apoptosis is induced through ligated death receptors, resulting in the generation of death inducing signaling complexes (DISCs) that result in caspase-8 and -10 catalytic activity (Kischkel et al., 1995; Walsh et al., 2003). While these pathways are initiated through distinct signals and pathways, they ultimately result in executioner caspase activity, and the orderly breakdown of the cell. This culminates in the generation of “apoptotic bodies,” fragments of the apoptotic cell that are readily engulfed by neighboring phagocytes; this engulfment of apoptotic cells prevents the release of cellular contents into the local tissue environment, and the ensuing inflammatory response that might result (Krysko et al., 2006).

Caspase-dependent apoptosis is relevant to a variety of diseases, as attested to by several thousand published manuscripts on the topic. Cancer cells have evolved numerous strategies to limit apoptotic sensitivity, and these include down-modulation of death receptors, expression of death receptor decoy molecules, altered expression of pro- or anti-apoptotic Bcl-2 family genes (Evan and Littlewood, 1998). Thus, significant effort has been placed into developing therapeutics that target intrinsic apoptosis regulators to resensitize tumor cells to apoptosis using drugs such as the Bcl-2 family BH3 domain mimetic ABT-737 (Garrison et al., 2012). The major problem such approaches face is that normal cells are also often sensitized by these therapeutics. Thus, identifying the pathways that promote tumor cell apoptosis without instigating the demise of normal cells is a difficult but essential goal. “Overactive apoptosis” has also been implicated in several degenerative diseases, including Alzheimer's disease, multiple sclerosis, Huntington's disease, and diabetes and others. As with cancer, modulation of apoptotic pathways may have therapeutic benefit only if they specifically target the cells that are implicated in the pathologic mechanism. While a challenging area, there is significant interest in the potential that apoptotic manipulation may yield clinical benefit in specific disease states.

## Programmed necrosis/necroptosis

An alternative form of programmed cell death has emerged that does not appear to require caspase activity that has been termed programmed necrosis, or necroptosis (Leist and Jaattela, 2001; Degterev et al., 2005). Originally observed to occur in response to artificial dimerization of the DISC adaptor protein FADD that is responsible for caspase-8 activation (Ashkenazi and Dixit, 1999), this form of cell death was observed to occur without the participation of fcaspases (Kawahara et al., 1998). This form of death was also found in TNF alpha-treated L929 cells cultured in the presence of pan-caspase inhibitors (Vercammen et al., 1998). Subsequent studies by the laboratory of Jürg Tschopp revealed that Jurkat cell lines deficient in components of the DISC are sensitive to TNF alpha-induced/caspase-independent cell death (Holler et al., 2000), and that this form of cell death requires the participation of the death domain containing serine/threonine kinase RIP1 (Stanger et al., 1995). This caspase-independent death was also observed in L929 following siRNA-mediated caspase-8 knockdown, implicating DISC regulation of this alternative form of cell death (Yu et al., 2004). This form of RIP1-dependent necroptosis was also observed to occur in T cells lacking FADD and caspase-8 activity (Bell et al., 2008; Ch'en et al., 2008), demonstrating that necroptosis may serve as a backup pathway to ensure the timely demise of lymphocytes following their clonal expansion (Bell and Walsh, 2009).

A major feature of DR-induced necroptosis is the involvement of RIP1. By developing “necrostatins,” inhibitors of TNF alpha-induced cell in FADD-deficient Jurkats (Degterev et al., 2005), Yuan and colleagues found that RIP1 catalytic activity is vital to “necroptosis” (Degterev et al., 2008). RIP3, a RIP kinase family member, is also required for TNF alpha-induced necroptosis, and forms a complex with RIP1 following apparent cross-phosphorylation of these two kinases (Cho et al., 2009; He et al., 2009; Zhang et al., 2009). The resulting RIP1/RIP3 containing “necrosome” is then able to promote downstream signals that promote the induction of necroptosis (Vandenabeele et al., 2010). Following its assembly, the necrosome leads to the RIP3-dependent phosphorylation of Mixed Lineage Kinase Domain-like Protein (MLKL), a process required for necroptosis (Sun et al., 2012). The mitochondrial protein phosphatase PGAM5 and mitochondrial fission factor Drp1 have also been found to participate in RIP3 mediated necroptosis, as well as that form of death induced by reactive oxygen species and calcium ionophores (Wang et al., 2012). Thus, a picture emerges in which, through stabilization of the RIP1/RIP3 necrosome, multiprotein complexes are formed that promote a necrotic form of cell death that occurs quite distinctly from caspase-dependent apoptosis.

While necroptosis and apoptosis are mechanistically and morphologically distinct processes, there is significant cross-talk between them. Importantly, apoptosis signaling opposes the stabilization of necrosomes following death receptor ligation. Both RIP1 and RIP3 are known targets of caspase-8 mediated cleavage (Lin et al., 1999; Feng et al., 2007), and thus it has been proposed that DISC assembly may prevent necrosome formation via caspase-8 (Declercq et al., 2009). Supporting this hypothesis, we observed that a caspase-8 resistant form of RIP1 introduced into RIP1-deficient Jurkats underwent necroptosis instead of apoptosis upon TNF alpha treatment (Lu et al., 2011). An alternative explanation holds that CYLD is the target of caspase-8 during the decision point between apoptosis vs. necroptosis (O'Donnell et al., 2011), although this model is not mutually exclusive from the aforementioned.

The existence of this alternative form of cell death prompts several questions, including the physiological relevance of the pathway and how it may be implicated in various disease states. While necroptosis has been primarily revealed under rather artificial conditions (e.g., using mutant cells or conditions with high-dose caspase inhibitors), its co-evolution with caspase-dependent apoptosis is suggested by the aforementioned cross-inhibition. Why might cells require necroptosis when apoptosis leads to an immunologically quiescent form of cell death? One potential explanation for this is that this form of death evolved to provide a “backup” pathway. Indeed, cowpox virus produces the factor CrmA, a potent apical caspase inhibitor capable of blocking apoptosis (Ray et al., 1992; Gagliardini et al., 1994). Several viral encoded genes are similarly produced to prevent apoptosis, and thus it is clear that inhibition of apoptosis is an important means viruses have exploited to avoid immune clearance. Interestingly, cytomegalovirus induces RIP3-mediated necroptosis via the interferon regulatory factor DAI (Upton et al., 2012). This virus also produces a protein called viRA, a protein that disrupts assembly of RIP1/RIP3 necrosomes and consequent necroptosis. Thus, while the physiological function(s) of necroptosis remains to be fully elaborated, it is clear that this cellular process has been around for a long time during evolution. It is likely that necroptosis may serve as an “Achille's heel” in tumor cells, and thus greater understanding of the process may reveal novel therapies for cancer therapeutics.

## Summary

Programmed cell death is clearly crucial to myriad biological processes, and much more is understood about the underlying mechanisms that modulate these with each passing day. This continued progress is important since programmed cell death is involved in numerous human diseases, and as described above and in other venues, targeting these mechanisms have or will have therapeutic benefit. Key areas of investigation are in understanding the specific involvement of apoptosis, necroptosis and other forms of cell death (e.g., “autophagic” cell death) in physiology and pathology. In addition, investigation into the mechanisms that regulate cell death, especially necroptosis and other under-explored forms of cell death, will be a significant endeavor. I envision that Frontiers in Cell Death and Survival will serve as an important venue for studies regarding apoptosis, necroptosis, and other forms of cellular death.
